# Supplementary material for: Synaptic representation of locomotion in single cerebellar granule cells
Source: eLife. 2015 Jun 17;4:e07290. doi: 10.7554/eLife.07290 (PMC4499793; doi:10.7554/eLife.07290)
Supplement: Source code 1. — Custom built software in Matlab (Mathworks). DOI: http://dx.doi.org/10.7554/eLife.07290.011 [file elife07290s001.zip › GCcode/MATLAB/neuronmodel/GrCModified/readme.html]

```
This is the README for cerebellar granule cell model code for the paper 

Shyam Diwakar, Jacopo Magistretti, Mitchell Goldfarb, Giovanni Naldi,
and Egidio D'Angelo.
Axonal Na+ channels ensure fast spike activation and back-propagation
in cerebellar granule cells.
J Neurophysiol (December 10, 2008).  doi:10.1152/jn.90382.2008
 
Implementation was done by Shyam Diwakar in Neuron.

In most neurons, Na(+) channels in the axon are complemented by others
localized in the soma and dendrites to ensure spike back-propagation.
However, cerebellar granule cells are neurons with simplified
architecture, in which the dendrites are short and unbranched and a
single thin ascending axon travels toward the molecular layer before
bifurcating into parallel fibers.  Following previous indications
(Magistretti et al., 2006; Goldfarb et al., 2007), Here we show that,
in cerebellar granule cells, Na+ channels are enriched in the axon,
especially in the hillock, but almost absent from soma and dendrites.
The impact of this channel distribution on neuronal
electroresponsiveness was investigated by multi-compartmental
modeling. Numerical simulations indicated that granule cells have a
compact electrotonic structure allowing EPSPs to diffuse with little
attenuation from dendrites to axon. The spike arose almost
simultaneously along the whole axonal ascending branch and invaded the
hillock, whose activation promoted spike back-propagation with
marginal delay (<200 micros) and attenuation (<20 mV) into the
somato-dendritic compartment. These properties allow granule cells to
perform sub-millisecond coincidence detection of pre- and postsynaptic
activity and to rapidly activate Purkinje cells contacted by the
axonal ascending branch.


Usage instructions:

Auto-launch from ModelDB or download and extract the archive.  Then
under:

----
MSWIN

run mknrndll, cd to the archive and make the nrnmech.dll.  Then double
click on the mosinit.hoc file.

----
MAC OS X

Drag and drop the GrC folder onto the mknrndll icon.  Drag and drop
the mosinit.hoc file onto the nrngui icon.

----
Linux/Unix

Change directory to the GrC folder. run nrnivmodl. Then type
nrngui mosinit.hoc
----
  
After launching the model, one may insert an current clamp electrode
to see the model response as in Fig.5A in the paper.

Choosing the excitatory mossy fibers and inhibitory fibers from
control panel, it is also possible to generate synaptic patterns as
reported in Fig.10 in the paper.  See the screenshot images in the
fig10 folder for details of how to set parameters for the different
subplots in figure 10.  For example setting the parameters as in
fig10/mf0p3_300.png leads to reproducing fig 10 second from top plot:


One may also modify each synaptic terminal individually through
"Companel.hoc" file.


 
Attention: 

The GrC model was adapted to have 4 excitatory and 4 inhibitory
synapses as observed in many cerebellar granule cells of rats. To
reconfigure please follow changes in the correspoding hoc files.

The model files were tested on Fedora 10 Linux and on MS-Win platforms
both running Neuron version 7.0.

Variable time step(pref DASPK method) works without issues while on
Current clamp protocol. While during synaptic activation, it is
recommended to turn it off.
```
